# Editorial: Signaling by Small GTPases in Metastatic Disease

**DOI:** 10.3389/fcell.2022.841572

**Published:** 2022-01-21

**Authors:** Peter J. Parker, Uday Kishore

**Affiliations:** ^1^ Francis Crick Institute and King’s College London, London, United Kingdom; ^2^ Biosciences, Brunel University London, Uxbridge, United Kingdom

**Keywords:** small GTPase, metastatic disease, cancer, superfamily, signalling

The superfamily of small GTPases are allosteric regulators par excellence, touching almost all corners of mammalian cell biology (Reiner and Lundquist, 2018). These GTPases sit on regulatory pathways impacting amongst other processes: membrane traffic, nuclear transport, cell division and cytoskeletal organisation. Their downstream effectors embrace a spectrum of functions from second messenger generators and protein kinases to compartmentalised binding platforms and transport chaperones. Given this breadth of action, it is perhaps not surprising that several family members have been implicated in the genesis and dissemination of cancers as exemplified by the Rho subfamily (Vega and Ridley, 2008).

Structurally, this family of GTPases retain a conserved core associated with their nucleotide binding function and for most superfamily members, but not all, there is an intrinsic GTPase activity which contributes to their dynamic behaviour switching from active (GTP bound) to inactive (GDP bound) states. Physiologically this switch is not a protein autonomous function governed simply by the GTP/GDP ratio, rather it is facilitated by exchange factors that unload GDP and enable reloading from the GTP dominated guanine nucleotide pool (Toma-Fukai and Shimizu, 2019), and GTPase-activating proteins that determine the rate of bound GTP hydrolysis and hence inactivation (Mishra and Lambright, 2016).

For the Ras subfamily of GTPases there has been long-standing interest in their driver roles in cancer, reflecting the penetrance of somatic gain-of-function mutations which occur in a breadth of different cancers. This has engendered a substantial literature on the targeting of these proteins (Moore et al., 2020) and it is not the intention to recapitulate this here. Rather this volume focuses on the wider membership of this class of regulators, where penetrant mutations are not generally associated with the GTPases themselves, rather involvement in cancer is evidenced by a combination of changes in expression, dysregulation of GTPase regulators (mutation and/or expression) and *a priori* consideration of specific roles for members of this family [see for example the Rho family (Haga and Ridley, 2016)]. Metastatic spread of disease is the major cause of mortality in cancer and it is this process in particular that is the focus of attention here. A multistep process that requires migration through basement membrane, intravasation into the circulation, extravasation to tissues and ultimately the lodging of the tumour in secondary sites (Madsen and Sahai, 2010). The extensive movement of tumour cells through “hostile” environments selects for many distinctive attributes amongst which is a demand on the reorganisation of the cytoskeleton, a well-established target of small GTPase action. Might intervention in these processes limit dissemination and the associated mortality?

Targeting these proteins is not trivial despite the presence of a druggable nucleotide binding pocket. This is largely the consequence of the very high affinity for GTP that generally precludes pocket-binding competitive inhibitors acting with any potency; the covalent inhibitors targeting the cys-mutant of Ras present an interesting exception to this generality (Lim et al., 2014). Alternative pockets on these proteins offer some opportunities, but their regulators, in particular the exchange factors, and some downstream effectors (e.g., protein kinases) have proven more tractable drugging challenges [for example, the PAKs (Semenova and Chernoff, 2017)]. Understanding these pathways, therefore, constitutes an important element in any attack on these processes.

In this volume, invited contributors review various aspects of the actions of some key representatives of the small GTPase superfamily. The validation of these as targets, their direct or indirect targeting and the potential for utility in the clinic are discussed. It is evident that much progress has been made in specific approaches to this general problem of intervention but nevertheless there is a great deal to do in exploiting our knowledge of these proteins to the benefit of patients. It is hoped that the articles in this volume will further stimulate efforts in this direction.

**OBITUARY**

**Dr Sunil Kumar Verma** (1974-2021)

**Figure:**
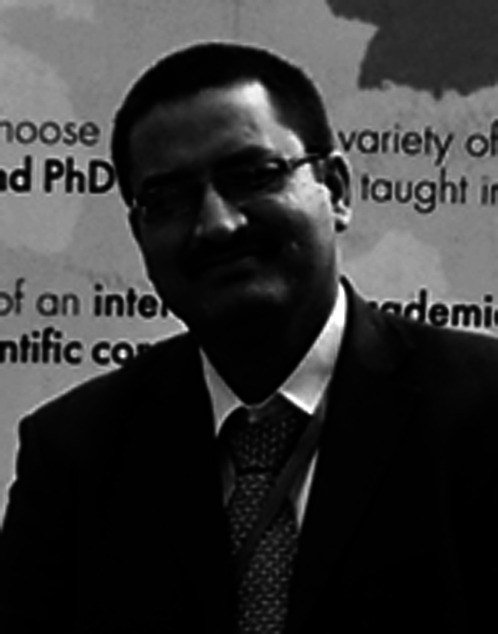


It is with great sadness that we mourn the death of Dr Sunil Kumar Verma, who was the main Guest Editor of this special issue. Sunil contracted SARS-CoV-2 infection and subsequently passed away due to COVID-19 pneumonia on 31^st^ May 2021 in the Indian City of Hyderabad, where he worked as a Principal Scientist within the Centre for Cellular and Molecular Biology (Council for Scientific and Industrial Research).

We came to know and work with Sunil while he was doing his DPhil (PhD) degree in Medical Oncology from the Weatherall Institute of Molecular Medicine, University of Oxford. He also worked in the laboratory of Prof Peter Parker at the ICRF, London, UK during the latter half of his DPhil degree.

Sunil was a well-known scientific figure in India for his contributions to the development of a DNA barcoding method, in collaboration with his mentor, Prof Lalji Singh. This application is used to great effect in wildlife forensics. Sunil had a stellar career at the national level that brought him several prizes and awards, including CSIR Technology Award, NRDC Meritorious Invention Award, Emerging Forensic Scientist Continental Award, and BioAsia Innovation Award. He was also the recipient of Lindau Fellowship, Commonwealth Scholarship, Max Plank Visiting Fellowship, and DAAD ambassadorship.

Sunil grew up in a small village called Tikri in the Indian state of Uttar Pradesh. He studied his BSc in Agriculture and Food Technology from the GB Pant University. All through his life, he remained a very humble, honest and hardworking person. He also carried out a range of outreach activities including publishing a series of poems on India’s topical issues.

Sunil will be sorely missed by his mentors, colleagues, family and friends. Rest in Peace!

Peter Parker, London, United Kingdom

Uday Kishore, London, United Kingdom
